# Cost-Sensitive Siamese Network for PCB Defect Classification

**DOI:** 10.1155/2021/7550670

**Published:** 2021-10-12

**Authors:** Yilin Miao, Zhewei Liu, Xiangning Wu, Jie Gao

**Affiliations:** ^1^School of Computer Science, China University of Geosciences, Wuhan 430078, China; ^2^Hubei Key Laboratory of Intelligent Geo-Information Processing, Wuhan 430078, China

## Abstract

After the production of printed circuit boards (PCB), PCB manufacturers need to remove defected boards by conducting rigorous testing, while manual inspection is time-consuming and laborious. Many PCB factories employ automatic optical inspection (AOI), but this pixel-based comparison method has a high false alarm rate, thus requiring intensive human inspection to determine whether alarms raised from it resemble true or pseudo defects. In this paper, we propose a new cost-sensitive deep learning model: cost-sensitive siamese network (CSS-Net) based on siamese network, transfer learning and threshold moving methods to distinguish between true and pseudo PCB defects as a cost-sensitive classification problem. We use optimization algorithms such as NSGA-II to determine the optimal cost-sensitive threshold. Results show that our model improves true defects prediction accuracy to 97.60%, and it maintains relatively high pseudo defect prediction accuracy, 61.24% in real-production scenario. Furthermore, our model also outperforms its state-of-the-art competitor models in other comprehensive cost-sensitive metrics, with an average of 33.32% shorter training time.

## 1. Introduction

**Figure 1:**
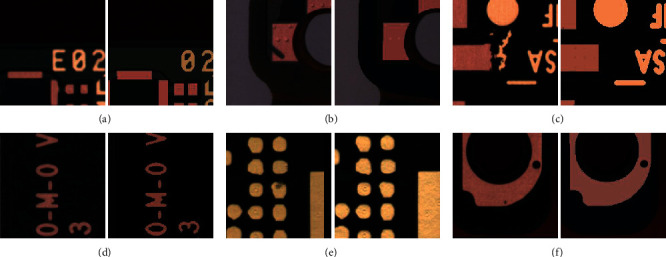
Examples of boards with different types of true and pseudo defects and their corresponding standard board images. (a) A board image with a true defect (missing character) and its corresponding standard board image. (b) A board image with a true defect (serious scratch) and its corresponding standard board image. (c) A board image with a true defect (short circuit) and its corresponding standard board image. (d) A board image with a pseudodefect (vague character) and its corresponding standard board image. (e) A board image with a pseudodefect (tiny dots on solder) and its corresponding standard board image. (f) A board image with a pseudodefect (tiny dots on copper) and its corresponding standard board image.

The fast development of low-cost electronic consumer devices such as home appliances and healthcare biosensors is creating need for printed circuit boards (PCB) at an unprecedented speed [1]. In such a fast developing and competitive field, it is important to find ways to reduce the cost of production. In the modern PCB manufacturing process, a large amount of hours of human work is spent on manually determining whether a defect proposed by automatic optical inspection (AOI) system is a true or a pseudo defect. True defects are serious defects which will cause the board to malfunction, while pseudo defects means defects which will not influence the performance of the PCB. A few examples of them are shown in Figure 1. Given that a true defect is much more harmful than a pseudo defect, this classification problem is cost-sensitive.

With the rapid development of PCB industry and the improvement of production technology, the traditional visual inspection method and AOI method can no longer meet current requirements. Therefore, new direction for defects classification technology has become urgent [2].

There is extensive research on automatic defect inspection, such as using thinning and flood-fill algorithms [3]. However, these studies emphasize on the detection of defects rather than improving the prediction accuracy for true defects. Some researchers used support vector machine (SVM) to classify between true and pseudo defects [4], but the accuracy could be improved. Moreover, very few of them focus on addressing the cost-sensitive nature of this problem. Most of the alarms raised by AOI machines are pseudo defects, while the defects that actually matter to the manufacturers and consumers are true defects. Failure to reduce true defects will be costly and could result in the loss of sales opportunities. Therefore, it is also important to consider cost-sensitive approaches besides improving defect classification accuracy.

The more advanced machine learning methods such as deep learning methods have been shown to have great performance on image classification and many other tasks [5–8]. As a cutting edge model of convolutional neural networks (CNN), siamese network is gaining a lot of attention with its ability to learn from pairs of images and extract useful information [9]. A siamese network is an artificial neural network that uses the same weights and identical architectures while working in tandem on two different input vectors to generate comparable output vectors [9]. Firstly, since relatively few images per class is sufficient for siamese networks to recognize those classes, e.g., one-shot learning [10], it is robust to cost-sensitive problems with small datasets. Secondly, it can learn from the semantic similarity [11] of pairs of images because it compares the embeddings of images after multiple layers. Furthermore, it puts previous unsused information such as the standard board images into use because it places the embeddings of same classes close together. We obtained our dataset from a real PCB factory. Our dataset contains the images of PCB boards with both true and pseudo defects, as well as standard board images. If we treat this problem as a traditional binary classification problem, the standard board images would not be used. Also, we use optimization algorithms to search for optimal thresholds after training, making the model cost-sensitive.

The goal of the proposed cost-sensitive siamese network (CSS-Net) is to firstly improve the overall classification accuracy on our dataset and secondly, to attain a high prediction accuracy for true defects while maintaining relatively reasonable prediction accuracy for pseudo defects. It would reduce human labour and has the potential of saving the industry a major part of the cost. Thirdly, parameters and training time of the model are reduced, so every batch of new data could be trained inside the factory more timely.

Our proposed model uses top layers of pretrained InceptionResNetV2 [12] as the feature-extractor and embed it with custom layers as the base model for the siamese network, and then we employ threshold-moving, a cost-sensitive method to maximize the accuracy for true defects, maintain high accuracy for pseudo defects, and save training time. The implementation of the proposed method is a model deployed after the AOI system, filtering and labeling most of the true and pseudo defects before the final human inspection, reducing human work hours.

**Figure 2:**
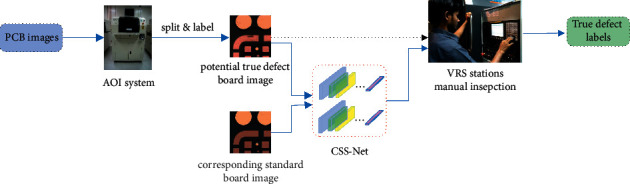
The usage scenario of our model deployed in a real PCB manufacturing facility.

The complete real-life process of our model implemented in a real PCB factory is as follows: Firstly, the images of every batch are taken by high-resolution cameras, prefiltered and labelled by AOI machines and split into images of different areas on a whole board. Then, each image with the prestored standard board image of that area was fed into our model to determine whether it contains true or pseudo defects, as shown in Figure 2. The workers at verify and repair system (VRS) stations only need to inspect the samples passed to them after the filtering of the model, saving a lot of human work hours, because most of the labelled images from AOI machines would be pseudo defects.

The rest of this paper is organized as follows.  Section 2 briefly introduces and discusses some related works.  Section 3 shows the structure and explanations of our proposed model.  Section 4 provides experiments and results as well as the description of our dataset.  Section 5 summarizes our conclusion and suggests the directions for future work.

## 2. Related Works

The most traditional method for PCB defect inspection is AOI. It is a traditional detection method using image processing and automatic control technology to detect PCB defects [13]. AOI is a noncontact online algorithm which is simple to design and employ. It can detect any kind of PCB defects which not only include short circuit defects but also include gaps, marks, and cavities. Most importantly, AOI systems are cheap and fast.

However, given that AOI method is pixel-based, its false alarm rate is particularly high. For instance, it is very common that random dust falls on the board, causing some pixels of the board to differ from the corresponding pixels on the standard board, which will cause the AOI system to raise alarms. Due to this obvious drawback, there are studies which use deep neural networks to classify PCB board defects. An automatic PCB defect detection system called Auto-VRS [14] was proposed by Deng et al. using AlexNet. They proved that Auto-VRS could reduce the false alarm rate of pseudo defects. In addition, Takagi et al. [15] proposed an approach for PCB defect detection and classification by extracting features from standard board images using CNN layers and then using an SVM classifier for classification. These approaches require preselection of feature maps, and the performance could be improved.

Alique et al. [16] proposed a neural network based model to predict the optimal cutting force in the milling process of industrial production, gaining decent results, which suggests that deep learning methods may have the value of application in the production of PCB. However, since our problem requires the classification of images, deeper CNNs are more suitable for our problem. In addition, Zhang et al. [17] proposed a modified ResNet model with an adjustment layer attached to the end. Dimitriou et al. [18] proposed an algorithm based on 3D convolutional neural networks (3DCNN) in order to predict upcoming events related to suboptimal performance in a manufacturing process. However, it focuses more on the prediction of upcoming events than scanning and filtering the existing defected boards. Schwebig and Tutsch [19] introduced an application strategy for combining a deep learning concept with an optical inspection system based on image processing. However, they used the already proposed DenseNet [20] which alone did not address the cost-sensitive nature of the problem.

Furthermore, Srimani and Prathiba [21] proposed a hybrid approach for feature reduction and classification, mainly to improve the performance of the prediction and classification. Firstly, they use genetic algorithm to select features, and then they use the deep neural network and Markov model for classification. There are also methods such as using SVM variations to solve the problem [22]. However, these methods require other metadata such as board production information apart from the images itself, adding complexity to their deployment processes.

There are also studies from an industrial standpoint, for example, Castaño et al. [23] proposed a procedure for evaluating the reliability of the data passed by the sensors; Villalonga et al. introduced a method to distribute computational resources for production [24], making an industry production system more robust. However, we focus more on the filtering of the seriously defected boards.

Combining CNN with transfer learning has been shown to have great performance on tasks such as image classification [25]. He et al. proposed a deep residual network in 2016, which greatly improved the capability of CNN to be deeper and its image classification accuracy [26]. Among CNN models, InceptionResNetV2 has excellent image classification accuracy [12], and siamese networks were used in various image classification problems [27, 28].

Cost-sensitive learning is becoming more and more popular among researchers. Studies on cost-sensitive methods with or without class-imbalanced datasets focus mainly on two aspects: the first is data preprocessing, of which the main method is sampling (by modifying the sample distribution of the training set to reduce the degree of data imbalance) [29] and the second is on the algorithm level, among which are mainly integrated learning, single-class classifier, and threshold-moving methods [30].

Although sampling methods have achieved good results on some datasets and can be well-combined with traditional machine learning methods [31], there are still drawbacks: oversampling can lead to overlearning of the classifier because it only repeatedly gets some artificially generated samples, and at the same time greatly prolongs the training time. In empirical studies, threshold-moving has great performance in cost-sensitive problems with class-imbalanced or class-balanced datasets [32, 33]. Thus, in our research, we use threshold-moving instead of sampling techniques.

Since we use threshold moving in our model, finding the optimal threshold is an important step. There are various optimization algorithms, such as simple multiobjective optimization based on cross-entropy (SMOCE) [34] proposed by Haber et al. We experimented with adaptive thresholds, and use some advanced optimization algorithms in pymoo [35] such as NSGAI-II [36], NSGA-III [37], RNSGA-II [38], and C-TAEA [39] to search for the optimal threshold values after the training of our model.

Scholars also proposed various methods to cope with the effects of environmental variables such as different illumination conditions in real-life productions. For example, Liu et al. proposed an illumination-invariant froth color measuring method (WDSPCGAN) [40] by solving a structure-preserved image-to-image color translation task. This would help keep the performance of the classification algorithms stable regardless of various lighting conditions. However, we do not need to consider the influence of illumination conditions because as shown in Figure 2, all of the pictures, we use, including defect board images with both true and pseudo defects, as well as standard board images, are taken inside standard AOI machines, with consistent environment parameters such as heat, humidity, and illumination.

## 3. Cost-Sensitive Siamese Network (CSS-Net)

### 3.1. Network Structure

The main notations in this paper and corresponding definitions are given in Table 1.

**Figure 3:**
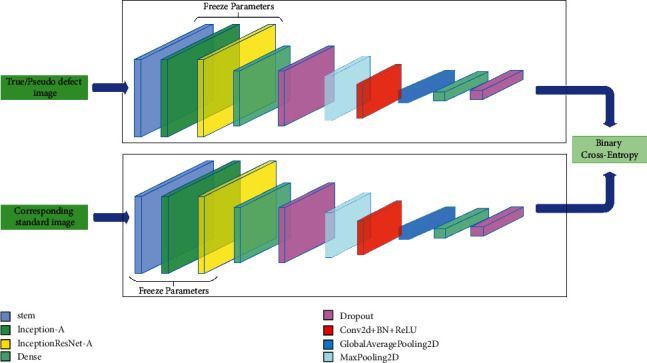
The overall architecture of the cost-sensitive siamese network (CSS-Net). The parameters for Conv2D are 32 (3, 3). The dropout rates are 0.2. The parameters for dense are 64 and 128.

Although large and complex networks perform well in classification problems, it comes at the cost of a much larger number of parameters, which slows down training. In our proposed model, we aim to reduce the total number of parameters as much as possible and improve the performance of the model at the same time.  Figure 3 shows the overall framework of this network.

**Figure 4:**
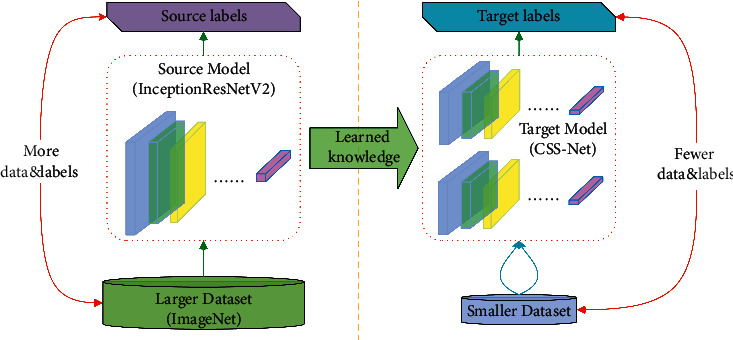
Model-based transfer learning.

The following layers are used in the main body of the network: fully connected layers (FC), batch normalization layers, rectified linear activation units (ReLU), max pooling operation layers (MaxPooling2D), average pooling operation unit (AvgPool), max pooling unit (MaxPooling2D), GlobalAveragePooling2D (flattens the input without affecting the batch size), and convolutional layers (Conv2D). We also implement transfer learning method, with the idea shown in the Figure 4.

**Figure 5:**
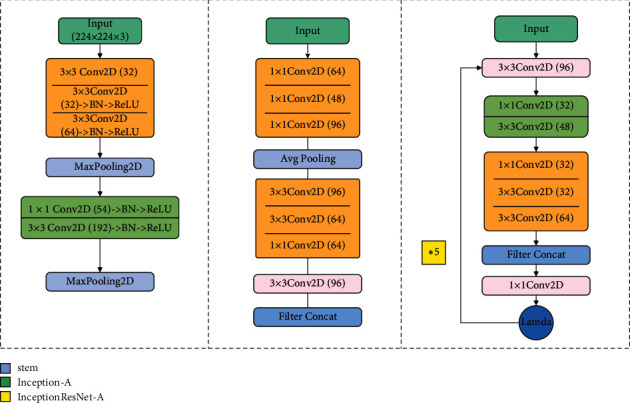
Structures of first three blocks in Figure 3.

The network is designed as follows: the base model is the already proposed InceptionResNetV2 [12]. It has great performance and contains a reasonable amount of parameters, and we use part of it as a feature extractor in order to save more training time and incorporate transfer learning for better generalization ability. We freeze the pretrained weights of the first three blocks of InceptionResNetV2 (“stem,” “Inception-A,” and “Inception-ResNet-A”) which were trained on the ImageNet dataset.  Figure 5 shows the composition of those blocks, after which we replace the rest of layers with custom layers and train the model on our dataset. The final step is to calculate the loss function using the Euclidean distance between the embeddings generated by pairs of images gone through the base model.

### 3.2. Explanation of the Structure

The overall steps of the algorithm are as follows (Algorithm 1).

To incorporate the transfer learning method in order to cope with the relatively small dataset, we freeze the weights of the top layers of the pretrained model and replace the rest of the layers with our custom-designed layers.

CNNs are layered architectures that automatically extract different features at different layers and generate hierarchical representations of them. This enables us to utilize a pretrained network (such as InceptionResNetV2) without its final layers as a fixed feature extractor for designated tasks.

**Figure 6:**
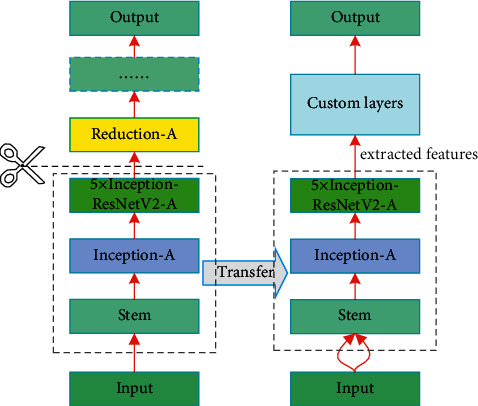
Pretrained layers as feature extractor.

In our model, we use the top layers of a pretrained network as a generic feature extractor, allowing us to train a new model on these features, making the model more stable and robust, and then we employ the threshold-moving approach in the end to make it cost-sensitive, as shown in Figure 6.

The using of pretrained weights in top layers instead of random initial parameters before fine-tuning enables our model a better starting point to cope with the relative small size of our dataset. Moreover, this allows the model to have a higher learning rate to reduce training time.

In the end, after experimenting with adaptive threshold values generated with designated *β* (introduced in Section 4) values, we use multiobjective optimization algorithms to search for the optimal threshold values instead. Our optimization target is to maximize *F* − score, *G* − mean, and sensitivity. The final result comes from the model after being threshold-adjusted using optimization methods.

Currently, we do not need to consider the influence of illumination conditions because all of the pictures we use, including defect board images with both true and pseudo defects, as well as standard board images, are taken inside standard AOI machines, with consistent environment parameters such as heat, humidity, and illumination. Our workflow, as shown in Figure 2, which is deploying a trained model of CSS-Net after AOI machines to significantly reduce the false alarms in order to save cost, occludes the necessity of adjusting for different environmental variables because in every batch of training, validating and testing data are generated in the same conditions. Workers at VRS stations only need to examine the filtered alarms. Not only can this one-time photo taking approach save time but also this rules out the possibility of random dust fallen onto the boards between machines to cause extra false alarms of those pseudo defects.

The motivations for techniques used in the model are as follows.

To improve the ability to have deeper lengths for models, in residual learning, the most important operation is identity mapping [26]. It adds the output from the previous layer to the layer ahead.

If **x** (identity) and ℱ(**x**) does not have the same dimension, a convolution operation would shrink its spatial resolution. The identity mapping is multiplied by a linear projection *W*_*s*_ to expand the channels of shortcut to match its resolution. This allows for the input **x** and ℱ(**x**) to be combined as input to the next layer.

(1) $$\begin{array}{c} y=ℱ\left(x,\left\{W_{i}\right\}\right)+W_{s}x. \end{array}$$

The convolutional layer is often connected to the batch normalization layer, which is an important means of reducing internal covariate shift, therefore preventing gradient disappearance or gradient explosion [41], which can cause instability in the network or even no further learning from the training data.

Before we pass on **x** to the next layer, we perform batch normalization transform to each minibatch:

(2) $$\begin{array}{c} h=f\left(g\cdot \frac{x-\mu }{\sigma }+b\right). \end{array}$$

Specifically, first we get a standard distribution with a mean of 0 and a variance of 1 through shift and scale transform:

(3) $$\begin{array}{c} \hat{x}=\frac{x-\mu }{\sigma }. \end{array}$$

Then, we transform it to a distribution with a mean of *b* and a variance of *g*^2^:

(4) $$\begin{array}{c} y=g\cdot \hat{x}+b. \end{array}$$

In standard batch normalization, shift parameter and scale parameter are defined as

(5) $$\begin{array}{c} \mu_{i}=\frac{1}{M}\sum x_{i},\\ \sigma_{i}=\sqrt{\frac{1}{M}\sum {\left(x_{i}-\mu_{i}\right)}^{2}}+ɛ. \end{array}$$

Batch normalization layer greatly improves the speed of training and the speed of convergence, as well as simplifies the process of tuning parameters and increases the effectiveness of classification.

Then, we use the activation function of ReLU (rectified linear unit), a layer that changes all negative activations (*y*_*i*_ < 0) to 0. This layer increases the nonlinear characteristics of the model and enables better training of deeper networks [42], compared to the other commonly used activation function such as logistic sigmoid.

(6) $$\begin{array}{c} \text{ReLU}\left(x\right)=\left\{\begin{array}{cc} 0 & \text{if }x\leq 0\\ x & \text{if }x>0 \end{array}\right.=\max\left\{0,x\right\}. \end{array}$$

We use the global average pooling [43] layer to replace the more traditional fully connected layer after the convolutional layers:

(7) $$\begin{array}{c} f_{i,j,k}=\underset{m}{\max}\left(w_{k_{m}}^{T}x_{i,j}\right). \end{array}$$

After the generated feature maps, we take the average of each feature map and feed the resulting vector directly into the softmax layer, instead of using a fully connected layer. This is more native to the convolution structure by enforcing correspondences between feature maps and categories, and it makes the model more robust to spatial translations of the input.

### 3.3. Loss Function

The Euclidean distances between the embeddings generated by pairs of images gone through the base model is fed into the loss function layer.

We firstly train our model with the contrastive loss function. The idea for this loss function is to make the distance between same class samples as small as possible and the distance between samples from different classes as large as possible. The contrastive loss function is defined as

(8) $$\begin{array}{c} \text{Loss}\left(W,Y,{\overset{⟶}{X}}_{1},{\overset{⟶}{X}}_{2}\right)=\left(1-Y\right)\frac{1}{2}{\left(D_{W}\right)}^{2}+\left(Y\right)\frac{1}{2}\max{\left(0,m-D_{W}\right)}^{2}, \end{array}$$

where *Y*=0 if the pair of samples ${\overset{⟶}{X}}_{1}$, ${\overset{⟶}{X}}_{2}$ belong to the same class and *Y*=1 if they belong to different classes. It will not update if the distance between ${\overset{⟶}{X}}_{1}$ and ${\overset{⟶}{X}}_{2}$ is greater than *m* in order to save time; if the distance between ${\overset{⟶}{X}}_{1}$ and ${\overset{⟶}{X}}_{2}$ is less than *m*, it will increase the distance between them to *m*.

The definition of binary cross-entropy is

(9) $$\begin{array}{c} \text{Loss}\left(\theta \right)=\log\prod_{i=1}^{N}p_{\theta }\left(y_{i}\right)=\log\prod_{i=1}^{N}\theta^{y_{i}}{\left(1-\theta \right)}^{\left(1-y_{i}\right)}=\sum_{i=1}^{N}\left[y_{i}\log\;\theta +\left(1-y_{i}\right)\log\left(1-\theta \right)\right],\\ \frac{\partial l}{\partial y}=-\sum_{i=1}^{n}\frac{{\hat{y}}_{i}}{y_{i}}-\frac{1-{\hat{y}}_{i}}{1-y_{i}}. \end{array}$$

**Figure 7:**
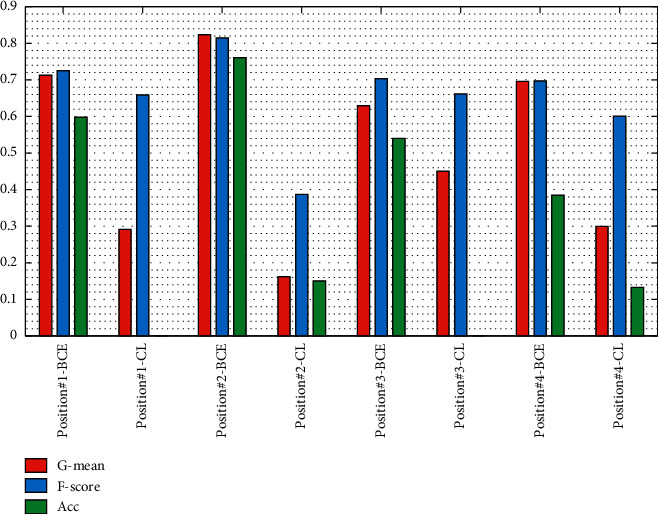
Results of different cutoff positions of InceptionResNetV2 as the feature extractor and different loss functions (threshold = 0.99). “BCE” denotes binary cross-entropy; “CL” denotes contrastive loss; position#1 denotes “block8_4_mixed”; position#2 denotes “block35_5_mixed”; position#3 denotes “mixd_6a”; position#4 denotes “block17_18_mixed” in InceptionResNetV2.

We used both loss functions and results show that the binary cross-entropy works better than the contrastive loss function on our dataset as shown in Figure 7.

## 4. Results and Discussion

### 4.1. Our PCB Defect Dataset

Our PCB defect dataset (hereinafter, referred to as PCB-ds) contains true defect board images, pseudo defect board images, as well as their corresponding standard board images. True defects consist of defects such as shape errors, color errors, character misprints, and broken solder resist areas, which will make the PCB malfunction. Pseudo defects include tiny dots on solder and tiny dots on copper, which will not influence its performance.

As shown in Table 2, the PCB-ds contains 22,500 pairs of true defect board images and their corresponding standard board images and 22,500 pairs of pseudo defect board images and their corresponding standard board images. The size of each image is 224^*∗*^244^*∗*^3. Of these, 90% are used as the training set, and 10% as the validation set. At the end of each epoch, the validation set is used to verify the performance. After training, an additional test set (consists of 2,500 true defect images with 2,500 corresponding standard board images; 2,500 pseudo defect images with 2,500 corresponding standard board images) was used to test the performance of the network. Finally, we adjust the threshold values and calculate cost-sensitive metrics.

### 4.2. Cost-Sensitive Evaluation Metrics

For the evaluation of the binary classifier, we introduce the confusion matrix, where TP indicates the prediction of true defects as true defects; TN indicates the prediction of pseudo defects as pseudo defects; FP indicates the prediction of pseudo defects as true defects; and FN indicates the prediction of true defects as pseudo defects, as shown in Table 3.

The most commonly used model evaluation metric is the accuracy (Acc). However, this metric could be misleading regarding cost-sensitive problems [44]. In such cases, other evaluation metrics should be considered in addition to the accuracy. Obviously, it is more costly to predict a true defect as a pseudo defect, which makes it a cost-sensitive problem, so we also calculate other secondary metrics: Sensitivity, Specificity, Precision, *G* − mean, and *F* − score to evaluate our proposed network. The definitions are as follows:

(10) $$\begin{array}{c} \text{Acc}=\frac{\text{TP}+\text{TN}}{\text{TP}+\text{TN}+\text{FP}+\text{FN}}, \end{array}$$

(11) $$\begin{array}{c} \text{True}-\text{Acc}=\text{Recall}=\text{Sensitivity}=\frac{\text{TP}}{\text{TP}+\text{FN}}, \end{array}$$

(12) $$\begin{array}{c} \text{pseudo}-\text{Acc}=\text{Specificity}=\frac{\text{TN}}{\text{FP}+\text{TN}}, \end{array}$$

(13) $$\begin{array}{c} \text{Precision}=\frac{\text{TP}}{\text{TP}+\text{FP}}, \end{array}$$

where sensitivity indicates the proportion of predicted true defects to all true defects; Specificity indicates the proportion of predicted pseudo defects to all pseudo defects; and Precision indicates the ratio of the number of predicted true defects to the actual number of true defects.

In addition, we use indicators like *F* − score and *G* − mean [45] to further verify the validity of our network, where the general *F* − score measurement is given in equation (13), and here, we take the *α*=1, i.e., equation (15), which we write it as *F*_1_, and the formula of *G* − mean is given in equation (16):

(14) $$\begin{array}{c} \text{F}=\frac{\left(\alpha^{2}+1\right)\text{precision}*\text{recall}}{\alpha^{2}\left(\text{precision}+\text{recall}\right)}, \end{array}$$

(15) $$\begin{array}{c} F-\text{score}=F1=\frac{2*\text{precision}*\text{recall}}{\text{precision}+\text{recall}}, \end{array}$$

(16) $$\begin{array}{c} G-\text{mean}=\sqrt{\text{sensitivity}*\text{specificity}}. \end{array}$$

The traditional *F* − measure or balanced *F* − score (F1 score) is the harmonic mean of precision and recall, and it takes both specificity and sensitivity into account and provides a balanced reflection of the accuracy of the algorithm.

The geometric mean (*G* − mean) measures the balance between classification performances on both true and pseudo defects [44]. A low *G* − mean would be an indication of a poor performance in the classification of the true defects even if the pseudo defects are correctly classified. This measure is important in order to avoid overfitting of pseudo defects and the underfitting of the true defects.

### 4.3. Determine the Network Parameters

**Figure 8:**
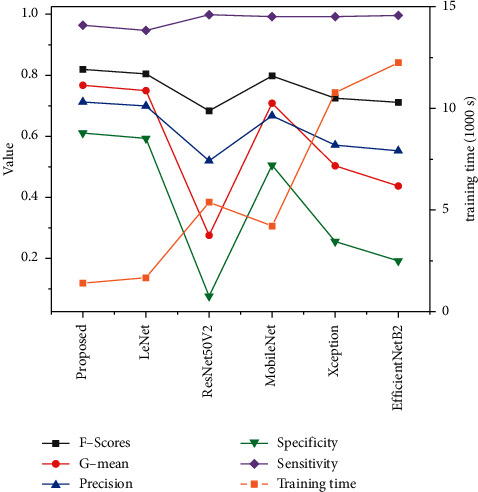
Results of different base models for siamese network structure.

To determine the base model of the siamese network, we test the performances of the siamese network with different base models, and the results are in Figure 8. It is clear that the selected InceptionResNetV2 is the most suitable base model, besides the scale of the original form of this model would be too large to train in a reasonable time.

To determine the cut-off position of the base model and loss function, as shown in results Figure 7, after experimenting with different ways of utilizing the layers of the base model as the feature extractor, we preserve the top layers from input to “block35_5_mixed,” which would generate best results.

### 4.4. Determine the Cost-Sensitive Threshold

It is relatively more important to prevent products with true defects than those with pseudo defects from going to the market. Therefore, our model requires higher accuracy on true defects than pseudo defects.

During training, each input would be a pair of true or pseudo defect image with its corresponding standard board image. When using the trained model, we will input each image with its corresponding standard board image too, as shown in Figure 2.

Because we employ the siamese network, if the prediction value is closer to 1, it means that the two images of input are more likely to be in the same class, and if the result is closer to 0, it means that the two images of input are less likely to belong to the same class.

To better illustrate the prediction process, for example, if the predicted value of a pair of true defect board image and its corresponding standard board image is less than the threshold, it is considered that they are not in the same class, which means it is a true defect, and the prediction would be correct (a pseudo defect can be considered equivalent to a standard board, for they would not cause the PCB to malfunction). Hence, if a false defect and its corresponding standard board is predicted greater than the threshold, they are considered to be of the same class, i.e., then the predicted result would be correct because the pseudo defect can be considered of the same class as the standard board and vice versa.

**Figure 9:**
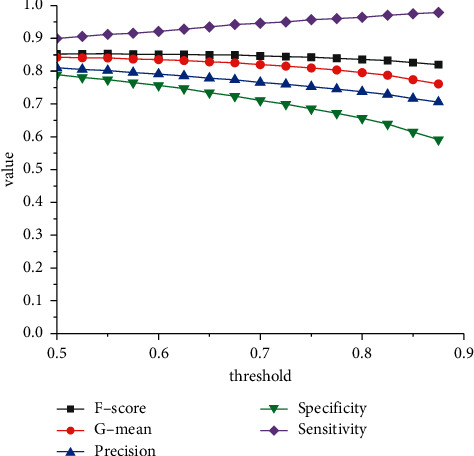
Illustration of threshold values.

As shown in Table 4, to make the model more sensitive to true defects, we change the default threshold 0.5 to greater values. As shown in Figure 9, there is a tendency that, as the threshold increases, the *F* − score and *G* − mean tend to decrease as specificity and precision decrease, while sensitivity tends to increase.

If we manually assign the importance of *F* − score, *G* − mean, and sensitivity with a coefficient *β*,

(17) $$\begin{array}{c} \text{objective}=\beta *F-\text{score}+\beta *G-\text{mean}+\left(1-2\beta \right)*\text{Sensitivity}, \end{array}$$

and set different *β* values, we will get Table 5.

However, the use of designated *β* values lacks reliability, for the same method should work on different batches of data, and in each batch, the importance of each metric could vary. So, we use multiobjective optimization algorithms to search for the optimal threshold values instead. Our optimization target is to maximize *F* − score, *G* − mean, and sensitivity. We can define our problem as follows:

(18) $$\begin{array}{c} \max f_{1}\left(x\right)=\text{Sensitivity}\left(x\right),\\ \max f_{2}\left(x\right)=F-\text{score}\left(x\right),\\ \max f_{3}\left(x\right)=G-\text{mean}\left(x\right),\\ \text{s}.\text{t}.\;0\leq x=\text{threshold}\leq 1. \end{array}$$

After our model is trained, *F* − score, *G* − mean, and sensitivity could be calculated at any given threshold values. Since it is not a linear objective function, we use various optimization methods based on genetic or evolutionary algorithms to search for near optimal values for the cost-sensitive threshold and get the results in Table 6.

### 4.5. Results and Comparisons

As shown in Table 7, by calculating the values of the metrics, the accuracy of CSS-Net for true defects is 97.60%, and the accuracy for pseudo defects is 61.24%, and it achieves an *F* − score and *G* − mean of 82.59% and 77.31%, respectively.

In order to compare our CSS-Net with state-of-the-art models more fairly, we also employ threshold-adjustment for those models after training as well since they were not originally designed to solve the cost-sensitive problem. As a result, the results of the state-of-the-art models shown in Table 7 are results of those models that have already been threshold-adjusted after training. We believe this would better highlight the contributions of CSS-Net to this problem.

Given that we emphasize on the sensitivity and cost-sensitive metrics of the model, the specificity and sensitivity values of those models would have converged to be roughly the same without threshold-adjustment since we are addressing this cost-sensitivity problem on a class-balanced dataset. Thus, we also used the same optimal threshold as CSS-Net. Moreover, the hyperparameters of all models such as batch size and learning rates are also set to the same, and iteration times are also the same unless some models take longer to converge. The network achieves the required results for both true and pseudo defect accuracy. Compared with other advanced and complicated networks, CSS-Net maintains high accuracy on both true-and pseudo defects while reducing training time by an average of 33.32%. Although our training time is larger than some simple networks, it is much smaller than the large-scale networks. And, the accuracies for both true and pseudo defects are higher.

Apart from the most important sensitivity metric, the *F* − score and *G* − mean metrics which are comprehensive metrics related to both true and pseudo defects classification accuracies are also taken into account because the practical significance each indicator represents is important. For example, although the accuracy for true defects is of more importance, the accuracy of pseud defects cannot be completely ignored in real production because special or novel defects predicted as pseudo defects may cause new types of malfunction which deserve to be documented. Although we focus more on the true defects, we still aim to increase both sensitivity and specificity as much as possible.

By combining the different evaluation metrics of each data, it can be concluded that our proposed network has a better overall performance on PCB defect classification and outperforms its state-of-the-art competitors, including Auto-VRS proposed by Deng et al. [14] and its base model AlexNet [46], the 2D version (because our dataset is two-dimensional) of 3DCNN appiled by Dimitriou et al. [18], and the DenseNet applied by Schwebig and Tutsch in their method [19], along with several popular generic models.

## 5. Conclusions and Future Work

To summarize, existing studies focus rarely on the cost-sensitive nature of the problem of PCB defect classification. Many of them require additional metadata other than the images of the boards. Moreover, most of them are not tested in a real-production scenario in PCB factories on a large scale, and the performance including accuracy and training time could be improved.

We propose a cost-sensitive siamese network (CSS-Net) with transfer learning technique to improve accuracy and cost-sensitive metrics of PCB defect classification. Extensive experiments on PCB-ds demonstrate that CSS-Net is superior to many existing methods. Our method achieves a true defect accuracy for 97.60%, a true defect accuracy for 61.24%, as well as an *F* − score and *G* − mean of 82.59% and 77.31%, respectively, in real-production scenario. The features of our model make it faster to train and deploy, and the cost-sensitive metrics of our model are better than its state-of-the-art competitor models, including Auto-VRS proposed by Deng et al. [14] and its base model AlexNet [46], the 3DCNN appiled by Dimitriou et al. [18], and the DenseNet applied by Schwebig and Tutsch in their method [19], along with several popular generic models, with an average of 33.32% shorter training time.

This method has the potential to save a substantial amount of workers' time and significantly reduce the cost of PCB boards. Also, thanks to its relatively short training time, every batch of new data of new designs of PCB boards can be trained in the factory timely once enough labels are accumulated. The overall true defect accuracy as well as comprehensive cost-sensitive measures such as *F* − score and *G* − mean which take into account both sensitivity and specificity values, are all improved. Meanwhile, the generalization capability of the model is also improved due to transfer learning. Combining this method with the traditional AOI machines would drastically reduce the human inspection requirement for the false alarms, which could potentially save a large part of the cost in PCB manufacturing.

We believe that, by exploring more novel approaches of cost-sensitive methods; improving the hyper-parameters; experimenting with more advanced structures; and improving the quality and quantity of our dataset, we could further improve the performance of our method, and these would be our main directions for future work.

## Figures and Tables

**Algorithm 1:**
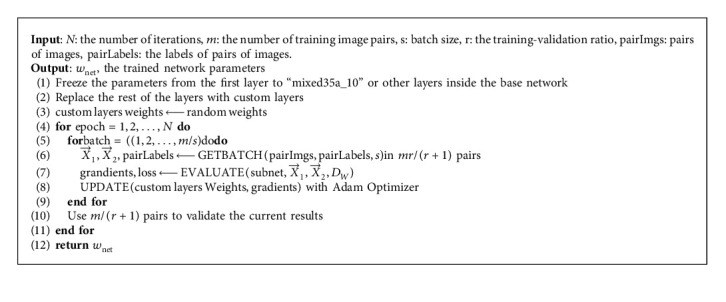
CSS-Net (cost-sensitive siamese network).

**Table 1:** Symbolic descirptions.

Symbol	Description
**x**	Input of a layer in the residual network
ℱ(**x**)	Output of a layer in the residual network
*ε*	Infinitely small number
*P*_*θ*_	Probability of *θ*
${\overset{⟶}{X}}_{1}$	Feature vector of the first label
${\overset{⟶}{X}}_{2}$	Feature vector of the second label
*D*_*W*_	Euclidean distance between ${\overset{⟶}{X}}_{1}$ and ${\overset{⟶}{X}}_{2}$
*μ*	Shift parameter
*σ*	Scale parameter

**Table 2:** Our PCB-ds dataset.

Dataset: PCB-ds			Type	Training set	Testing set	Validation set
Image information of dataset	Width	224	Positive	20,250	2,500	2,250
	Height	224				
	Channels	3	Negative	20,250	2,500	2,250
	Number	50,000				

**Table 3:** Confusion matrix.

		Real class	
		True defect	Pseudo defect
Predicted	True defect	True positive (TP)	False positive (FP)
Class	Pseudo defect	False negative (FN)	True negative (TN)

**Table 4:** Results of different threshold values.

Threshold	*F* − score	*G* − mean	Precision	Specificity	Sensitivity
0.450	0.8539	0.8481	0.8259	0.8136	0.8840
0.475	0.8532	0.8451	0.8170	0.8000	0.8928
0.500	0.8525	0.8426	0.8102	0.7892	0.8996
0.525	0.8526	0.8411	0.8052	0.7808	0.9060
0.550	0.8533	0.8404	0.8017	0.7744	0.9120
0.575	0.8516	0.8370	0.7959	0.7652	0.9156
0.600	0.8514	0.8351	0.7912	0.7568	0.9216
0.625	0.8509	0.8325	0.7856	0.7468	0.9280
0.650	0.8497	0.8286	0.7787	0.7344	0.9348
0.675	0.8494	0.8258	0.7734	0.7240	0.9420
0.700	0.8467	0.8202	0.7659	0.7108	0.9464
0.725	0.8445	0.8154	0.7598	0.6996	0.9504
0.750	0.8426	0.8099	0.7525	0.6852	0.9572
0.775	0.8394	0.8034	0.7454	0.6720	0.9604
0.800	0.8357	0.7956	0.7373	0.6564	0.9644
0.825	0.8327	0.7877	0.7290	0.6392	0.9708
0.850	0.8262	0.7741	0.7166	0.6144	0.9752
0.875	0.8202	0.7611	0.7057	0.5916	0.9792

**Table 5:** The optimal threshold value and corresponding objective value for designated *β* values.

*β*	Threshold	Objective	*β*	Threshold	Objective
0.1	0.8852	0.9420	0.3	0.8405	0.8717
0.15	0.8400	0.9231	0.35	0.8452	0.8536
0.2	0.8400	0.9060	0.4	0.8457	0.8363
0.25	0.8402	0.8889	0.45	0.8450	0.8190

**Table 6:** Results of optimization algorithms.

Algorithm	Threshold	*F* − score	*G* − mean	Sensitivity
NSGA-II [36]	0.8544	0.8257	0.7729	0.9760
NSGA-III [37]	0.8542	0.8259	0.7731	0.9760
RNSGA-II [38]	0.8536	0.8259	0.7731	0.9760
C-TAEA [39]	0.8576	0.8244	0.7702	0.9764

**Table 7:** Results of different networks.

Network	*F* − score	*G* − mean	Precision	Specificity	Sensitivity	Training time (s)
CSS-Net	**0.8259**	**0.7731**	**0.7158**	**0.6124**	**0.9760**	776
Auto-VRS [14]	0.7903	0.7258	0.6813	0.5600	0.9408	238
3DCNN-2D [18]	0.7535	0.7127	0.6792	0.6004	0.8460	589
AlexNet [46]	0.7767	0.6985	0.6615	0.5188	0.9404	**247**
NasNet [47]	0.6446	0.2345	0.4953	0.0596	0.9228	1,214
ZFNet [48]	0.7783	0.7126	0.6723	0.5496	0.9240	385
MobileNet [49]	0.8187	0.7636	0.7087	0.6016	0.9692	820
EfficientNetB2 [50]	0.7846	0.7175	0.6753	0.5500	0.9360	2,235
DenseNet [20]	0.7910	0.7082	0.6676	0.5168	0.9704	2,722
ResNet [26]	0.7727	0.7274	0.6880	0.6004	0.8812	2,025

## Data Availability

The dataset used in this study was obtained from a real PCB factory and could not be published. The other data supporting the results could be obtained by contacting the corresponding author.
